# Return-to-Play Practices Following Hamstring Injury: A Worldwide Survey of 131 Premier League Football Teams

**DOI:** 10.1007/s40279-019-01199-2

**Published:** 2019-10-08

**Authors:** Gordon Dunlop, Clare L. Ardern, Thor Einar Andersen, Colin Lewin, Gregory Dupont, Ben Ashworth, Gary O’Driscoll, Andrew Rolls, Susan Brown, Alan McCall

**Affiliations:** 1 Arsenal Performance and Research Team, Arsenal Football Club, London, UK; 2 Edinburgh Napier University, Sport Exercise and Health Science Research Group, School of Applied Sciences, Edinburgh, UK; 3 Department of Medical and Health Sciences, Division of Physiotherapy, Linkoping University, Linkoping, Sweden; 4 Department of Sports Medicine, Norwegian School of Sport Sciences, Oslo Sports Trauma Research Centre, Oslo, Norway; 5 Lewin Sports Injury Clinic, London, UK; 6 Medical Department, French Football Federation, Paris, France; 7 Performance Department, AC Sparta Prague Football Club, Prague, Czech Republic; 8 Sport Science and Medical Department, Bristol City Football Club, Bristol, UK

## Abstract

**Purpose:**

Return-to-play (RTP) is an on-going challenge in professional football. Return-to-play related research is increasing. However, it is unknown to what extent the recommendations presented within research are being implemented by professional football teams, and where there are gaps between research and practice. The purposes of this study were (1) to determine if premier-league football teams worldwide follow a RTP continuum, (2) to identify RTP criteria used and (3) to understand how RTP decision-making occurs in applied practice.

**Methods:**

We sent a structured online survey to practitioners responsible for the RTP programme in 310 professional teams from 34 premier-leagues worldwide. The survey comprised four sections, based on hamstring muscle injury: (1) criteria used throughout RTP phases, (2) the frequency with which progression criteria were achieved, (3) RTP decision-making process and (4) challenges to decision-making.

**Results:**

One-hundred and thirty-one teams responded with a completed survey (42%). One-hundred and twenty-four teams (95%) used a continuum to guide RTP, assessing a combination of clinical, functional and psychological criteria to inform decisions to progress. One-hundred and five (80%) teams reported using a shared decision-making approach considering the input of multiple stakeholders. Team hierarchy, match- and player-related factors were common challenges perceived to influence decision-making.

**Conclusions:**

General research recommendations for RTP and the beliefs and practices of practitioners appear to match with, the majority of teams assessing functional, clinical and psychological criteria throughout a RTP continuum to inform decision-making which is also shared among key stakeholders. However, specific criteria, metrics and thresholds used, and the specific involvement, dynamics and interactions of staff during decision-making are not clear.

**Electronic supplementary material:**

The online version of this article (10.1007/s40279-019-01199-2) contains supplementary material, which is available to authorized users.

## Key Points

**Table:** 

A range of clinical, functional and psychological criteria were assessed across four phases of a RTP continuum by premier-league football teams worldwide.
Absence of pain, hamstring strength, training load and functional performance/sport-specific tests were the most frequently reported top three criteria assessed.
There was no consistent information given to advance knowledge on specific metrics and thresholds for criteria.
Despite consistent involvement reported of medical staff in a shared decision-making process, there were differences in the reported involvement of science staff, coaches and players.
While faced with several challenges, teams typically achieved the criteria they set.

## Introduction

A disconnect between sport science and medicine research with practice is often cited by professional football teams [1, 2] despite an evidence-led approach being recommended as gold-standard to optimise high-performance outcomes [3–5]. Return-to-play (RTP) is often discussed and debated in professional football, and RTP-related research is increasing rapidly. In particular, an expert-led 2016 consensus statement [6] and two subsequent Delphi surveys focussing on professional football and RTP from hamstring muscle injury (the most common injury in football) [7, 8] have provided some key recommendations for improving RTP. However, it is unknown if the recommendations are followed in practice, and if not, what barriers could be preventing their adoption. Translation of research into the practical setting has a great potential to develop and deliver new information that can enhance RTP practices [9, 10]. However, we must first determine if RTP research is being translated into practice and identify if, where and why gaps exist.

The 2016 RTP consensus statement [6] recommended that: (1) RTP should be viewed as a continuum rather than a single event at the conclusion of rehabilitation, and follow through to ‘return-to-performance’, (2) objective markers should guide RTP progression and (3) practitioners should follow a shared decision-making process including key stakeholders (e.g. staff, coaches, players). While both of the football-specific Delphi surveys centred on RTP after hamstring injury [7, 8] and recommended key criteria and objective markers, including clinical tests to assess tissue healing (e.g. pain, flexibility, strength), measures of training-load (e.g. Global position satellite (GPS) systems), functional sport-specific performance tests (e.g. repeated-sprint ability, acceleration/deceleration, maximal sprints) and psychological readiness.

While previous consensus and Delphi recommendations aimed to provide practitioners with a number of specific tests and proposed cut-off values to help inform RTP decision-making (e.g. 0–10% difference in active/passive straight leg raise or eccentric hamstring strength when compared to pre-injury benchmark values and/or contralateral limb) [6–8], it is unclear how criteria, tests and thresholds are actually used in the practical setting (if at all). Additionally, while it is recommended that the RTP decision-making process should be shared among key stakeholders, details of what this looks like in professional football have yet to be provided.

To determine if current research recommendations are being translated into practice, and if not, where and why gaps potentially exist, the purposes of this study were (1) to determine if premier-league football teams worldwide follow a RTP continuum, (2) to identify RTP criteria used and (3) to understand how RTP decision-making occurs in applied practice.

## Methods

### Participants

Between 24th-October-2017 and 20th-March-2018 (2017–2018 season), 310 professional football teams from 34 premier-leagues worldwide were invited to participate. The purpose and procedure of the online survey was explained, and a web-link provided. We requested the survey be completed by the person/s of the science and sports medicine team responsible for the design and implementation of the RTP programme. Institutional ethical review board approval was granted by Edinburgh Napier University (SAS/00014). Confidentiality and anonymity were detailed before consenting to participate.

We sent a maximum of three reminder emails over a 6-week period from the first email invitation. A follow-up email was also sent if there were missing data. If the question/s remained unanswered, it was excluded from analysis.

### Survey

The survey design and construction followed recommendations on the design and development of surveys [11]. The survey underwent 3 rounds of piloting (for content validity and usability) with 12 experienced applied researchers/practitioners working in professional football (but not from any teams invited to participate). Twelve modifications resulted: four items deleted and eight added. The survey was originally developed in English and translated (using cross-cultural adaptation process recommended by World Health Organisation (WHO) [12] into French, Spanish, German, Italian, Portuguese (+Brazilian Portuguese) and Japanese. The survey was administered online (Novi Survey, http://novisurvey.net).

Respondents were asked to consider their RTP practices during the previous season for a typical football-related hamstring muscle injury (time-loss 18 days) [13] when answering all questions in the survey. There were 29 questions (10 closed, 19 open) (Appendix 1 in Supplementary material) organised into four sections, which were adapted (by the steering committee and through the piloting process) for use in football but based on a RTP continuum model:

1. Return-to-high-speed running (RTRun)—the period between hamstring injury occurring and the player being cleared to run on-field and progresses to high-speed running
2. Return-to-train (RTTrain)—when the player was allowed to return to on-field unrestricted training
3. Return-to-play (RTPlay)—when the player was cleared to return to competitive match-play with the team (whether selected or not)
4. Return-to-performance (RTPerf)—when the player returned to pre-injury levels of performance (or higher).

Each section comprised four parts *(except RTPerf, which only considered parts 1 and 2):

1. Use of RTP continuum, criteria used to progress each phase (5 closed and 7 open questions)
2. Achieving desired criteria before moving to next phase (3 open questions)
3. Decision-making process to progress each phase (3 closed questions)
4. Challenges (i.e. barriers) faced when progressing from one phase to the next (3 open questions).

### Survey Analyses

The survey closed on 31st-April-2018. Raw data were exported to Microsoft Excel. To ensure content analysis accuracy, native speakers skilled in translation verified the translation accuracy of answers to open-ended questions where necessary. We used a cross-sectional design and analysed results descriptively according to the checklist for reporting results of internet e-surveys (CHERRIES) [14].

To evaluate the importance of specific criteria, and corresponding test/tool for clearance to the next RTP phase, we assigned rankings [15–18]. For each continuum phase, respondents specified and ranked in order of importance (1st–3rd) the criteria they considered to determine RTP progression. For each phase, criteria ranked in 1st, 2nd and 3rd position were reported as a frequency (%) of total responses.

To analyse the open-ended questions, we used inductive content analysis [19] following a three-stage process [20–22]. We treated survey answers as standalone meaning units, unless they contained more than one self-definable point, in which case, each meaning unit was considered and separated. Responses with insufficient information were excluded. For each section of the survey, meaning units generated from responses pertaining to each question were listed, before being compared for similarities and organised into raw data themes. Raw data themes were grouped for each question into larger and more general themes/categories in a higher order concept [21]. We continued refining the data until theoretical saturation [23].

To enhance our confidence in interpreting the data, two independent authors (GD and AM) read the lists of meaning units at least twice [24]. They discussed meaning units, categories and themes at each stage to reach a consensus regarding data accuracy and clarity. Sample data sets were re-examined by a third independent researcher, blind to the research aims, to audit the assigned categories and themes to ensure they accurately reflected the standalone meaning units [25].

## Results

Three-hundred and four teams consented to participate. One-hundred and one (33%) teams failed to respond having consented to participate; 72 (23%) teams were excluded due to incomplete survey responses. In total, 131 (42%) teams completed the survey and were included in analysis. A full list of participating confederations with affiliated countries and premier-leagues surveyed is presented in Table 1. The positions of respondents were: club doctor (61 teams); physiotherapist (33 teams); strength and conditioning coach (26 teams); sports scientist (9 teams) and manual therapist (2 teams).

**Table 1:** Details of the response rate among invited premier-leagues (confederation and country)

Football Confederation	Union of European Football Associations (UEFA)	Asian Football Confederation (AFC)	South American Football Confederation (CONMEBOL)	Confederation of North, Central American and Caribbean Association Football (CONCACAF)	Confederation of African Football (CAF)	Anonymous
Survey Response Breakdown (Invited/Responded/Included)	(225/129/86)	(50/40/25)	(9/9/9)	(23/12/7)	(3/3/3)	(N/A/115/1)
**Associated Premier Leagues Surveyed**	Austria (2/1/1)	Australia (10/10/7)	Argentina (3/3/3)	North America (20/9/5)	South Africa (3/3/3)	Unknown (115/1)
	Belgium (8/5/3)	China (5/3/0)	Brazil (3/3/3)	Mexico (3/3/2)		
	Croatia (7/1/0)	India (1/1/0)	Uruguay (3/3/3)			
	Denmark (10/9/6)	Iran (1/1/0)				
	England (20/20/13)	Japan (18/11/9)				
	France (21/11/8)	Qatar (12/12/8)				
	Germany (14/5/2)	UAE (2/2 /1)				
	Holland (13/7/2)	Saudi Arabia (1 /0/0)				
	Israel (1/1/1)					
	Italy (20/17/13)					
	Norway (16/13/6)					
	Portugal (18/8/8)					
	Russia (4/2/1)					
	Scotland (12/8/7)					
	Spain (17/10/8)					
	Sweden (14 /1/0)					
	Switzerland (8/4/2)					
	Turkey (10/6/4)					
	Poland (1/0/0)					
	Greece (9/0/0)					

### Return-to-Play Continuum

In total, 124/131 premier-league teams (95%) reported following a return-to-play continuum model. Of the 124, 27 (21%) teams did not report following a ‘return-to-performance’ phase (RTPerf).

### Criteria Used During RTP

**Fig. 1:**
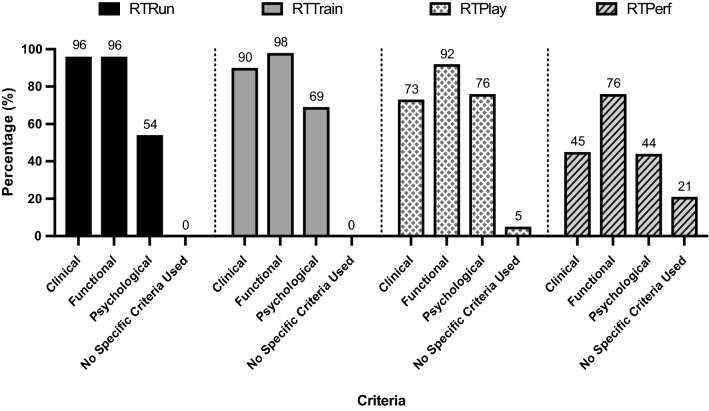
Criteria used by teams at each phase of the return-to-play continuum to guide progression

For both RTRun and RTTrain phases, all teams used a criterion-based approach. At RTPlay 7 (5% of 131) teams reported they did not use specific criteria to determine a player’s clearance. This increased to 27 (21%) teams at RTPerf (Fig. 1). Table 2 provides an overview of the specific criteria used by teams and the level of importance given to guide progression at each phase of the continuum.

**Table 2:** The frequency (%) of reporting top three criteria across the RTP continuum

Continuum Phase	RTRun			RTTrain			RTPlay			RTPerf		
Criteria	1st	2nd	3rd	1st	2nd	3rd	1st	2nd	3rd	1st	2nd	3rd
Absence of pain	57*	21	27*	12	8	4	7	5	4	2	2	2
Hamstring strength	17	40*	24	22	29*	18	3	6	5	8	8	0
Hamstring flexibility	8	21	15	2	1	3	1	1	2	1	2	0
Functional performance/assessment	5	6	8	11	18	19	24	18	14*	6	5	7
Staff subjective appraisal	3	3	6	8	3	5	7	4	5	11	14	15*
Psychological readiness	5	3	9	2	2	8	6	7	13	11	14	11
Training load monitoring	1	2	3	39*	25	20*	41*	38*	14*	33*	21*	15
Other (e.g. medical imaging, time)	5	5	5	2	5	2	0	1	2	0	0	1
**Total (%)**	**100**	**100**	**97**	**98**	**92**	**80**	**89**	**79**	**58**	**72**	**64**	**50**

Totals (%) for each ranking position across each phase are denoted by bold

*The most frequently reported criteria for that RTP phase. Please note that in phases and/or individual ranking positions where totals do not reach 100%—the remaining % represents the proportion of blank responses

### Frequency with Which Criteria were Met Before Progression

**Fig. 2:**
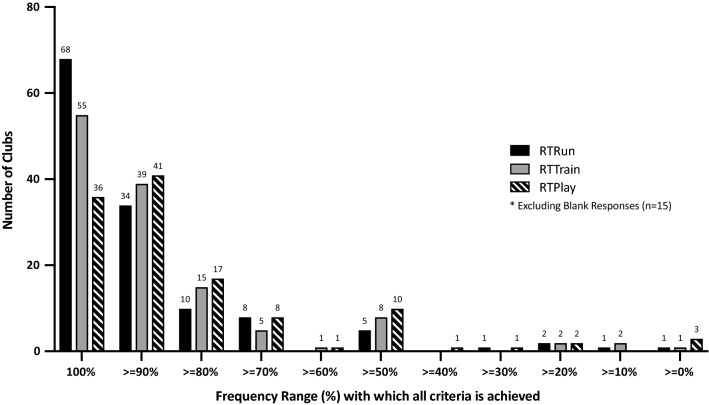
The frequency which teams reported achieving all the criteria they set across each phase of the return-to-play continuum

We included 378 out of 393 (96%) responses, i.e. 131 responses × 3 main RTP phases. Across each phase, the response rate of teams was 130/131 (99%); 128/131 (98%) and 120/131 (92%) for RTRun, RTTrain and RTPlay, respectively. When returning to RTRun, a frequency of 100% was reported by 68 (52%) teams (i.e. all intended criteria were met before the player was cleared to progress by 68 teams). By comparison, 55 teams at RTTrain and 36 at RTPlay reported with 100% frequency to always successfully meeting the criteria set. The frequency range (%) with which teams successfully reported to achieving all of the intended criteria is displayed in Fig. 2.

### The RTP Decision-Making Process

We analysed 389 out of 393 (99%) responses. Per phase, 131/131 (100%) teams responded for both RTRun and RTTrain while 127/131 (97%) answered at RTPlay. Overall, 105 (80%) teams use a shared decision-making approach involving at least two people. Table 3 represents the contribution of key staff members to decision-making based on the position (i.e. medical or science) of the practitioner who completed the survey (Table 3).

**Table 3:** The contribution of key staff members to decision making across the phases of the return-to-play continuum based on the perspective and position held by the responding practitioner

Stakeholder/s involved in the decision-making process to inform progression	Stakeholder involvement when reported by Medical Team (*n* = 96)			Stakeholder involvement when reported by Science Team (*n* = 35)			Difference in response between Medical Team versus Science Team responses		
	RTRun (*n*)	RTTrain (*n*)	RTPlay (*n*)	RTRun (*n*)	RTTrain (*n*)	RTPlay (*n*)	RTRun (%)	RTTrain (%)	RTPlay (%)
**Medical staff **	**94**	**94**	**83**	**35**	**34**	**31**	**98* vs* 100**	**98*vs* 97**	**87*vs* 89**
*Club doctor*	74	79	68	27	27	25	77 *vs* 77	82 *vs* 77	71 *vs* 71
*Physiotherapist*	78	75	58	33	28	25	81 *vs* 94	78 *vs* 80	60 *vs* 71
**Science staff**	**39**	**53**	**53**	**30**	**34**	**31**	**41*vs* 86**	**55*vs* 97**	**55* vs* 89**
*Strength & conditioning coach*	33	45	44	28	32	30	34 *vs* 80	47 *vs* 91	46 *vs* 86
*Sport scientist*	16	27	28	12	15	16	17 *vs* 34	28 *vs* 43	29 *vs* 46
*Sport psychologist*	1	1	1	0	1	0	1 *vs* 0	1 *vs* 3	1 *vs* 0
**Coaches and management**	**11**	**30**	**73**	**8**	**19**	**30**	**11*vs* 23**	**31*vs* 54**	**76*vs* 86**
*Manager*	8	17	40	2	6	12	8 *vs* 6	18 *vs* 17	42 *vs* 34
*Coach (technical staff)*	4	19	52	7	14	26	4 *vs* 20	20 *vs* 40	54 *vs* 74
**Player**	**48**	**51**	**51**	**18**	**23**	**24**	**50*vs* 51**	**53*vs* 66**	**53*vs* 69**

Stakeholder groups are denoted by bold with the staff members affiliated to each stakeholder group presented in italics

### Challenges Influencing Decision-Making

Challenges relating to team hierarchy (e.g. pressure from management) were regarded the most likely to influence practitioner decision making (27% of challenges cited) (Table 4). As a player transitioned to RTPlay, match related factors were more prominent. We excluded 132 responses (100 due to a blank response; 32 due to an error in the cross-cultural translation in the Spanish version that was not picked up during final piloting—the question could have been misinterpreted). Twelve teams stated that challenges were not applicable as every player must have met all criteria before being cleared.

**Table 4:** The challenges faced when helping a player return to play

Challenge	RTRun	RTTrain	RTPlay	Total
Hierarchical	29	38	42	109
Match-related	28	30	39	97
Player-related	32	29	24	85
Team-related	18	13	26	57
Rehabilitation programme-related	12	19	10	41
Other challenges	6	9	6	21
No challenges encountered	6	7	8	21

Hierarchical challenges, e.g. pressure from management/internal staff agreement; Match-related challenges, e.g. importance of upcoming fixture(s)/phase of season; Player-related challenges, e.g. compliance to progress, pressure to progress/return; Team-related challenges, e.g. existing squad depth/other injuries; Rehabilitation programme-related challenges, e.g. time constraints, isolated decision making; Other challenges, e.g. language barriers, limited resources/facilities; External factors, e.g. media, sponsors, agents

## Discussion

Our structured survey revealed that the majority of premier-league teams surveyed (124; 95%) used a continuum approach to guide RTP following hamstring injury using a combination of clinical, functional and psychological criteria. Clinical criteria were most common at RTRun and RTTrain, while functional criteria were consistently assessed across all phases. Across the later phases of the RTP continuum, greater focus was placed on the assessment of psychological readiness. Eighty percent of clubs adopted a shared decision-making process with at least two people involved at any one phase. Despite myriad of challenges being perceived to influence decision-making, teams often met the criteria that they set to progress through the RTP continuum.

### RTP Continuum in Premier League Football Teams Worldwide

Based on our sample of premier-league teams worldwide, the majority (124; 95%) assessed criteria over a continuum to guide RTP following hamstring injury. Of 124 teams, 102 (78%) reported assessing criteria at the four specified phases; RTRun, RTTrain, RTPlay and RTPerf. Of the remaining 29 teams, 22 (17%) implemented a criteria-based approach at RTRun, RTTrain and RTPlay, but not RTPerf. Unfortunately, the teams did not provide sufficient details for us to confidently report why this was the case; however, of the minimal feedback we did receive, it was specified that they believed the RTPlay phase should be where the player is also considered to be back to full performance.

Seven (5%) teams did not follow a RTP continuum and did not explain why. Our findings provide preliminary support (at least in our sample) that general research recommendations and practice align in that the majority of team practitioners view RTP from the point of injury until at least returning to play and most through until returning to desired performance. Our RTP continuum differs from the one specified in the 2016 consensus statement. In particular, we had specified an additional phase early in rehabilitation (RTRun). Football (and sport in general) and research are constantly evolving, and the application of a continuum framework within and between sports may need to be adapted to the specific needs of those monitoring and controlling the overall RTP process. Therefore, models such as the RTP continuum may need to be adaptable to suit these needs and research should consider this also.

### Criteria were Widely Used to Guide RTP but Highly Varied Across Premier League Teams

Team practitioners used a combination of clinical, functional and psychological criteria to guide RTP following a hamstring muscle injury. Multifactorial and criteria-based rehabilitation programmes are advocated in research to support RTP decision-making [26–28]. Such a criteria-based decision approach provides practitioners with an individualized approach to RTP that integrates quantifiable assessment (objective and subjective) to systematically progress rehabilitation. Criteria-based approaches may reduce re-injury risk and improve player performance and availability of footballers [26, 29]. In our survey, we asked respondents to specify their top three most important criteria used at each of the RTP phases (Table 2) with the aim of uncovering some consistently used criteria, metrics and thresholds that could inform current practice and guide future research.

#### Criteria to Progress to RTRun

While over seven different criteria were represented at this phase, absence of pain and hamstring strength were the two most frequently reported top three criteria used to inform progression to RTRun. Absence of pain (reported frequency; 1st–57%, 2nd–21%, 3rd–27%) aligns with perceptions previously presented in the research literature [7, 8, 30]. Within our survey, emphasis appeared to be placed on the absence of pain during clinical evaluation (e.g. on palpation, or strength and flexibility tests) and/or following functional performance testing (e.g. running mechanic drills, low-moderate speed running) which is similar to the RTP Delphi survey of football experts by van der Horst and colleagues [7]. In a recent systematic review [31] of criteria used to inform rehabilitation progression and RTP clearance following hamstring strain injury, it was highlighted that progression was typically only permitted within pain-free limits. The presence of localized discomfort on palpation following return-to-play may increase the risk of hamstring re-injury in athletes [32]. Remaining pain free during rehabilitation has also been challenged with the suggestion that it may unnecessarily prolong rehabilitation, thereby increasing the injury burden [31]. Additionally, athletes’ subjective ratings of pain poorly quantify progress within rehabilitation following hamstring injury [33]. Therefore, there does not appear to be any clear and confident recommendations on the role of ‘absence of pain’ prior to RTRun or in general throughout RTP process.

Relative to other recorded criteria, hamstring strength was also more frequently reported by practitioners as a top three criteria at RTRun (reported frequency; 1st–17%, 2nd 40% and 3rd–24%). There is an important consideration with strength, however, which was identified in the Delphi surveys of van der Horst and colleagues [7] and Zambaldi and colleagues [8], in which ‘strength’ can encompass a variety of types and evaluations (e.g. eccentric, isometric, imbalance between legs and within legs). Yet what specific components of strength should inform RTP progression remain unclear. In the Zambaldi et al. [8] consensus, it was agreed that full hamstring strength is essential to for a safe RTP. However, in contrast, the experts in the Delphi survey of van der Horst and colleagues [7] did not reach consensus, with experts unable to agree if eccentric strength should be used as a criterion, although they did agree that other contraction types should not be used as criteria for RTP. Unfortunately, our survey respondents did not provide sufficient information on the types of hamstring strength they tested as criteria. In 2014, Tol and colleagues [28] showed that normalisation of isokinetic strength was not necessary for successful hamstring RTP in professional footballers, while a 2017 systematic review [32] recommended the opposite: that hamstring strength could be a useful criterion during hamstring RTP. However, the systematic review was not specific to professional football only and specificity of population is arguably necessary. Since then, scientific studies (e.g. cohort studies) are building that question the utility of hamstring strength and specifically isokinetic cut-values as progression criteria for hamstring RTP [34–36]. However, it should be noted that these studies are concerned with the RTPlay phase and to our knowledge no studies have investigated the role of strength prior to returning to high-speed running.

#### Criteria to progress from RTRun to RTTrain

To inform progression to RTTrain, despite a variety of top three criteria being reported, training load (reported frequency; 1st–39%, 2nd–25% and 3rd–20%) and hamstring strength (1st–22%, 2nd–29%, and 3rd–18%), were the most frequently reported criteria by practitioners. Hamstring strength was discussed in the previous section. The higher reported frequency of training load monitoring is consistent with the perceptions of medical practitioners in UEFA Champions League [17] and FIFA national teams [15] where training load was highlighted as one of the top criteria for injury prevention. It is currently unclear how training load relates to re-injury risk or specifically, muscle/hamstring re-injury, if at all. While only expert opinion, it has been recommended to maintain ‘high control’ over running loads (and speeds) during this rehabilitation phase with particular consideration given to the progression of speed and player characteristics, e.g. position, style of play [37]. We discuss training load as to how it might relate to the RTP in the following RTTrain to RTPlay phase.

#### Criteria to Progress from RTTrain to RTPlay

To inform RTPlay decision-making, training load was again a criteria more frequently considered by practitioners (1st–41%, 2nd–38% and 3rd–14%). Existing RTP recommendations advocate achieving GPS benchmarks based on player/position-specific match metrics (e.g. max speed, high-speed running distance, sprint number) are important to ensuring readiness to RTPlay [7, 8]. Stares and colleagues [38] recently reported that longer RTPlay (to progressively develop greater weekly and total training loads) was associated with reduced risk of re-injury in Australian rules footballers. Specifically, achieving running loads above peak values prior to the injury resulted in an extra ~ 10 days (31.6 ± 10.8 days vs. 21.6 ± 2.5 days) missed. We should be aware that the time to progress through RTP phases is an ongoing risk assessment whereby an extra 10 days missed could be the difference between two to three matches (in elite contemporary football) and potentially up to nine points.

The finding that performance/sport specific field testing was one of the more frequently reported criteria at this phase was not surprising (1st–24%, 2nd–18% and 3rd–14%). This criterion should theoretically allow practitioners to assess the player’s readiness to load the injured muscle as required during progression to activities with higher demands as seen at RTTrain and RTPlay. Performance during on-field testing was considered to be a ‘vital’ criteria in determining RTP clearance by the football experts [7]. A carefully planned RTP programme that addresses all aspects of the game may be important for restoring functional performance levels while minimizing the risk of re-injury [26, 39]. However, further research is needed to validate functional tests to guide RTPlay decisions.

#### Criteria to Determine When Players Have Returned to Performance

While the majority of premier league teams followed a RTP continuum approach, RTPerf was the one phase that 21% teams highlighted that they did not follow with anecdotal feedback suggesting that they believed players should be back to desired performance levels upon RTPlay. Defining what represents the desired performance level is important and to our knowledge this has not yet been achieved in the research literature. The criteria for RTPerf proposed in the 2016 consensus statement [6] stated that this phase may be categorized by personal best performance or expected growth as it relates to performance. In the professional football setting this likely refers to match-related metrics related to physical, technical, tactical and cognitive qualities.

As with RTTrain and RTPlay, training load was one of the most frequently reported criteria (1st–33%, 2nd–21%, 3rd–15%), yet little is currently known about training load and RTPerf. Given that the majority of a starting player’s in-season loading is derived from match play (i.e. typically 2 games/week), the inability to maintain training load throughout rehabilitation has been suggested as a risk factor for re-injury and may contribute to the high rate of ‘early’ recurrences (< 2 months) observed following RTPlay [40, 41]. Normalization of training loads comparable to the team was not achieved until after RTPlay in Australian rules football [42], while footballers returning to play were at increased risk of subsequent injury for up to 12 weeks [43]. Accordingly, extending player monitoring/observation beyond RTPlay may represent an interesting aspect to assess during the RTPerf phase, as recommended by Stares et al. [43] to not only ensure pre-injury performance benchmarks are being achieved but also as a tertiary-level injury prevention strategy. However, this represents only one preliminary study and in a different sport.

#### Other Considerations Regarding Criteria

Psychological criteria were highlighted in the global criteria used by team practitioners (Fig. 1) and specified as important to consider in the research literature [44–47] as well as the previous Delphi surveys conducted in elite football [7, 8]. Psychological readiness was infrequently reported by practitioners. In view of the modifiable nature of psychological factors/traits, it has been recommended in research that psychological factors should to be assessed from the time of injury [48]. While limited in football, expression of positive psychological responses across rehabilitation (e.g. higher motivation, low fear of re-injury) has been associated with successful return-to-sport (i.e. RTPlay in our study) outcomes within a variety of different athletic populations [44, 49, 50]. Few practitioners specified which psychological readiness tool they used (if they used any formal evaluation). This may be due to a lack of well-validated instruments to measure psychological readiness and may explain the relatively low accumulated points. Research is urgently needed to validate and evaluate the effectiveness of psychological readiness questionnaires for professional footballers.

### What does RTP Decision-Making Look Like in Practice?

A shared decision-making approach was used by 80% of premier-league teams surveyed. This is an encouraging finding as low-quality internal communication may be associated with (re)injury rates and reduced player availability [17, 51, 52]. Only eight (6%) teams reported using isolated decision-making across all continuum phases. Eighteen (14%) teams used a combination of isolated and shared approaches to guide rehabilitation progression.

Medical staff (club doctors and physiotherapists) were most frequently consulted throughout the decision-making process. Traditionally regarded as the gatekeepers of the RTP decision, medical staff clearly hold a prominent role within the decision-making practices of clubs. In 96 teams (73%), medical staff were the lead practitioner responsible for the RTP programme. Across each phase of the RTP continuum, ≥ 87% of teams consulted with at least one medical practitioner (Table 3).

While medical staff involvement in decision-making across all RTP continuum phases was reported by both medical and science practitioners surveyed (Table 3), their perceptions as to how other stakeholder groups are involved in decision-making throughout RTP differed. Specifically, medical staff reported less involvement of science and coaching staff across all phases of the continuum and for players at RTTrain and RTPlay compared to when science staff answered the survey. We cannot answer why this is, as potential bias for respondents placing greater emphasis on the involvement of their own discipline should then have also been evident in the responses of science staff, yet this was not the case. Our results raise an important question about how staff are actually involved in the RTP continuum process. Despite an initial encouraging finding that the RTP decision-making is shared among stakeholders, the inconsistency found in the composition raises some potential concerns about the specific dynamics of the communication among staff.

### Achieving the Criteria Set Across the RTP Continuum

Premature RTP has been suggested as a possible risk factor for re-injury [41, 53–55]. Throughout the RTP continuum, surveyed practitioners highlighted encountering various challenges capable of influencing their decision-making (Table 4). When progressing through the RTP continuum following hamstring injury, team practitioners reported that there were occasions when the player did not meet all of criteria set (Fig. 2). However, these occasions were not common. Typically, teams met the criteria they set ≥ 90% of the time, yet, the variations demonstrate the reality of the practical setting where it is not possible to achieve this all of the time.

Each injury case must be assessed individually, based on a risk assessment. Accordingly, the risk associated with accelerating a player’s RTP to ensure availability for a decisive fixture may be more readily accepted in the case of the key 1st team player as opposed to the promising youth team prospect—who might be afforded a longer RTP timeframe to reduce reinjury risk. While surveyed teams predominantly displayed a high degree of success in achieving criteria, this finding reflects only one muscle-group (hamstring). Therefore, we do not know if this is representative of rehabilitation across other muscle-groups or injury types.

### Limitations

An inherent limitation of survey-based research is its lack of external validity owing to low response rates. One hundred and thirty-one (42%) of 310 invited teams completed the survey. Accordingly, caution should be exercised when interpreting or generalizing these results, as the extent to which they characterise the perceptions and practices of the non-responding teams is unclear. How these findings extend to other levels of competition (professional vs. amateur), genders, different age groups (senior-level vs. academy-level) and other muscle-groups or injury-types is also unknown and warrants consideration in future research. Representing current opinion (level 5 evidence), we acknowledge our findings may change with emerging evidence and paradigm shifts. Therefore, the perceptions and practices of practitioners should be re-evaluated in the future, based on new research recommendations. While sampled clubs appear to display a high degree of success in meeting their outlined criteria, a perceived limitation (although not a specific focus of our survey) could be that we did not ask practitioners to elaborate on instances where RTP was accelerated without achieving criteria. It is not known if, in these instances, re-injury occurrences predominantly occurred. We also acknowledge that survey responses correspond only to the perceptions and practices of science and medical practitioners responsible for the return-to-play programme. It is possible that responses could vary according to the position of the stakeholder surveyed while the perceptions of other key stakeholders’ groups involved in decision-making (e.g. managers, players) were not considered. We could not compare cultural differences as participating clubs from different confederations/leagues were not equally represented. Further investigation adopting techniques capable of facilitating a more comprehensive picture (e.g. qualitative focus groups, individual interviews etc) of how specific metrics and thresholds inform return-to-play decision-making is required.

## Conclusion

Professional football teams assessed a range of clinical, functional and psychological criteria to support decision-making on whether or not to progress a player at four key phases (in our survey—RTRun, RTTrain, RTPlay, RTPerf) of the RTP process. While a wide variety of criteria were used, the most frequently reported criteria to progress to high-speed running were absence of pain and hamstring strength. When returning to full training, hamstring strength and training load were more frequently reported than any other criteria. The transition to full match-play revealed training load and functional performance/sport specific tests as the more frequently reported criteria. However, insufficient information regarding the specific metrics and thresholds used for these RTP criteria highlight that the lack of clear research guidelines also appears to be an issue in the practice of professional football teams. Encouragingly, professional football teams reported using a shared decision-making process throughout the entire RTP process. However, the proportion of those involved at each phase was only consistent for medical staff (club doctors and physiotherapists). The specific involvement of sport science staff, coaches and players was less clear and should be explored in more detail. While there were instances where team practitioners reported progressing players without meeting all of the criteria they set, these instances were not overly frequent. Practitioners can be encouraged that despite facing a number of challenges (including but not limited to, hierarchical, match and player related), professional football practitioners can still meet the criteria they set a large proportion of the time.

## Electronic supplementary material

Below is the link to the electronic supplementary material.

Supplementary material 1 (PDF 1499 kb)
