# Supplementary material for: Return-to-Play Practices Following Hamstring Injury: A Worldwide Survey of 131 Premier League Football Teams
Source: Sports Med. 2019 Oct 8;50(4):829–40. doi: 10.1007/s40279-019-01199-2 (PMC7069905; doi:10.1007/s40279-019-01199-2)
Supplement: Supplementary file 1 — Supplementary material 1 (PDF 1499 kb) [file 40279_2019_1199_MOESM1_ESM.pdf]

Electronic Supplementary Material

This supplementary material has been provided by the authors to give readers additional information about their study.

Supplement to: Gordon Dunlop[1,2], Clare Ardern[3], Thor Einar Andersen[4], Colin Lewin[5,] Gregory Dupont[6], Ben Ashworth[1*], Gary O'Driscoll[1], Andy Rolls[,7], Susan Brown[2], Alan McCall[1,2]. Is there a gap between research and practice in Return to Play for elite football teams? A worldwide survey of premier league teams. Sports Medicine

Corresponding Author

Alan McCall

Arsenal Football Club

Bell Ln, London Colney

Hertfordshire, AL2 1DR

Tel: +33 651748266 - Fax: +33 320887363

Email: amccall@arsenal.co.uk

Affiliations:

[1] Arsenal Football Club, Performance and Research Team, London, UK

[2] Edinburgh Napier University, Sport Exercise & Health Science Research Group, School of Applied Sciences, Edinburgh, UK

[3] Linkoping University, Department of Medical and Health Sciences, Division of Physiotherapy, Linkoping, Sweden

[4] Oslo Sports Trauma Research Centre, Department of Sports Medicine, Norwegian School of Sport Sciences, Oslo, Norway

[5] Lewin Sport Injury Clinic, London, UK

[6] Medical Department, French Football Federation, Paris, France

[7] Bristol City Football Club, Sport Science and Medical Department, Bristol, UK

Progression criteria during return to play following a hamstring injury in professional football

The purpose of this survey is to determine the perceptions and practices put in place by the medical and sport science departments of premier league football teams worldwide regarding the criteria used to progress players throughout the return to play process following a typical hamstring time loss muscle injury of 18 days (Esktrand et al., 2016).

Please answer the survey based on your perceptions and practices during the 2016-17 season. The survey should take approximately 15 minutes to complete.

A report of the overall findings will be sent to each participating team. In accordance with the 1998 Data Protection Act all completed individual responses will be treated confidentially and anonymised.

Any publications and presentations concerning this survey will consist of overall results only and no identifying information will be shown or disclosed. The overall findings of this survey could be presented in congress and/or published in scientific articles.

1. I agree to participate

○ Yes ○ No

Progression criteria during return to play following a hamstring injury in professional football

Respondent Demographics

2. To be completed by the lead practitioner responsible for the return to play programme

Position held

League of club

Club (Optional)

If applicable, state the confederation cup competition you competed in last season (e.g. UEFA Champions league, Copa Liberatadores)

Please state which round of this competition you reached (e.g. group stages, knockout stages)

Progression criteria during return to play following a hamstring injury in professional football

Definitions

Injury Diagnosis

Typical hamstring time loss muscle injury - 18 days (Ekstrand et al., 2016)

Definition of Rehabilitation Phases

1. From Injury to Return to High Speed Running - The period between the injury occurring and the player being cleared to run on-field and progress to high speed running

2. Return to Run to Return to Training - When you allow the player to return to on-field unrestricted training with the first team

3. Return to Training to Return to Play - When the player is cleared to return to competitive match-play with the first team (whether selected or not)

4. Return to Play to Return to Performance - When the player has been deemed to return to pre injury levels of performance (or higher)

Progression criteria during return to play following a hamstring injury in professional football

Section 1: Return to High Speed Running

The period between injury occurring and the player being cleared to run on-field and progress to high speed running

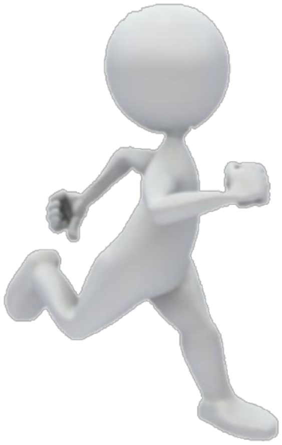

3. **Do you consider any of the following criteria to determine a players' clearance to return to high speed running?** *Select as appropriate*

- [ ] Clinical
- [ ] Functional (e.g. physical, movement)
- [ ] Psychological
- [ ] None, we do not use any specific criteria at this stage
- [ ] Other

4. **In order of importance, being as specific as possible, specify up to 3 criteria you use to decide a player is cleared to return to high speed running (e.g. pain, flexibility, X% of pre injury hamstring eccentric or isometric strength etc)**

1. ____________________
2. ____________________
3. ____________________

5. **With respect to the criteria you mentioned above, please specify (in corresponding order to Q4) which tool or test do you use to measure this (e.g. VAS-100 scale, Asking H-test, force plate etc)**

1. ____________________
2. ____________________
3. ____________________

6. **In general, please specify how often do you clear a player to return to high speed running without meeting all of the specific criteria you set?**

Frequency (%)

7. What are the main challenges (if any) you face that would lead you to clear a player to return to high speed running before they have met the criteria you have set?

Please specify below

Challenges [ ]

8. At this stage, who is typically involved in the decision-making process to release or clear a player to return to high speed running?

Select as appropriate

- [ ] Manager
- [ ] Coach (technical staff)
- [ ] Club Doctor
- [ ] Physiotherapist
- [ ] Player
- [ ] Sport Scientist
- [ ] Strength and Conditioning Coach
- [ ] Other

9. If dealing with a quadricep, calf or adductor muscle injury, is there anything you would change or add with respect to the criteria, tools or tests you implement during this phase of rehabilitation?

Please specify below

Adductor [ ]

Quadricep [ ]

Calf [ ]

Progression criteria during return to play following a hamstring injury in professional football

Section 2: Return to Train

When you allow the player to return to on-field unrestricted training with the first team

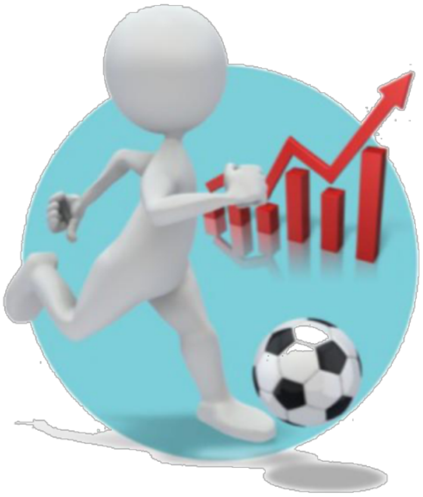

10. Do you consider any of the following criteria to determine a players' clearance to return to train? *Select as appropriate*

- [ ] Clinical
- [ ] Functional (e.g. physical, movement)
- [ ] Psychological
- [ ] None, we do not use any specific criteria at this stage
- [ ] Other

11. In order of importance, being as specific as possible, specify up to 3 criteria you use to decide when a player is cleared to train (e.g. X% of average or maximal high-speed running performed in a match, within X% of pre injury hamstring eccentric strength, limb symmetry index >X% etc)

1. ______
2. ______
3. ______

12. With respect to the criteria you mentioned above, please specify (in corresponding order to Q11) which tool or test do you use to measure this (e.g. global positioning system (GPS), nordbord, isokinetic dynamometry etc)

1. ______
2. ______
3. ______

13. In general, please specify how often do you clear a player to return to training without meeting all of the specific criteria

you set?

Frequency (%)

14. What are the main challenges (if any) you face that would lead you to clear a player to return to training before they have met the criteria you have set?

Please specify below

Challenges

15. At this stage, who is typically involved in the decision-making process to release or clear a player to return to train? *Select as appropriate*

- Manager
- Coach (technical staff)
- Club Doctor
- Physiotherapist
- Player
- Sport Scientist
- Strength and Conditioning Coach
- Other

16. If dealing with a quadricep, calf or adductor muscle injury, is there anything you would change or add with respect to the criteria, tools or tests you implement during this phase of rehabilitation?

Please specify below

Adductor

Quadricep

Calf

Progression criteria during return to play following a hamstring injury in professional football

Section 3: Return to Play

When the player is cleared to return to competitive match-play with the first team (whether selected or not)

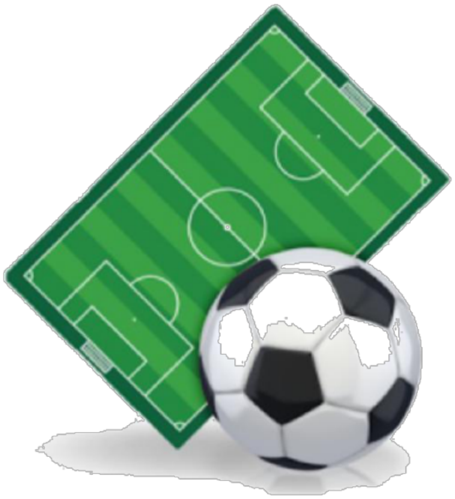

17. Do you consider any of the following criteria to determine a players' clearance to return to play? *Select as appropriate*

- [ ] Clinical
- [ ] Functional (e.g. physical, movement)
- [ ] Psychological
- [ ] None, we do not use any specific criteria at this stage
- [ ] Other

18. In order of importance, being as specific as possible, specify up to 3 criteria you use to decide when a player is cleared to return to play (e.g. achieved X% of players worst case match scenario for sprint distance, exposure to maximal speed X times, horizontal force mechanics etc)

1.
2.
3.

19. With respect to the criteria you mentioned above, please specify (in corresponding order to Q18) which tool or test do you use to measure this (e.g. global positioning system (GPS), speed gates, radar gun etc)

1.
2.
3.

20. In general, please specify how often do you clear a player to return to play without meeting all of the specific criteria you set?

Frequency (%)

21. What are the main challenges (if any) you face that would lead you to clear a player to return to play before they have met the criteria you have set?

Please specify below

Challenges

22. At this stage, who is typically involved in the decision-making process to release or clear a player to return to play? *Select as appropriate*

- [ ] Manager
- [ ] Coach (technical staff)
- [ ] Club Doctor
- [ ] Physiotherapist
- [ ] Player
- [ ] Sport Scientist
- [ ] Strength and Conditioning Coach
- [ ] Other

23. If dealing with a quadricep, calf or adductor muscle injury, is there anything you would change or add with respect to the criteria, tools or tests you implement during this phase of rehabilitation?

Please specify below

Adductor

Quadricep

Calf

Progression criteria during return to play following a hamstring injury in professional football

Section 4: Return to Performance

When the player has been deemed to return to pre injury levels of performance (or higher)

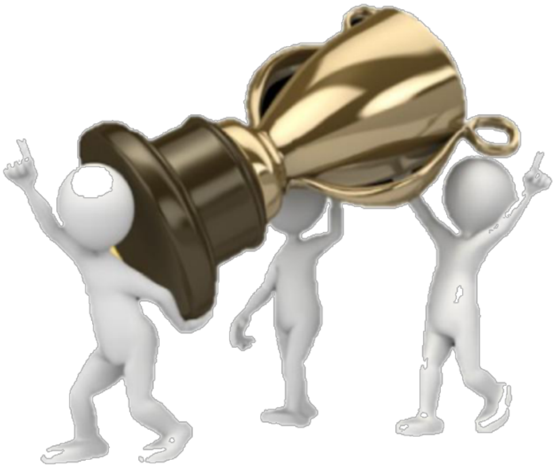

24. Once cleared to return to play, do you continue to monitor the player to assess when/if pre-injury performance levels (or higher) have been achieved?

- Yes
- No

25. Do you consider any of the following criteria to determine a players' return to pre-injury levels of performance (or higher)?

Select as appropriate

- Clinical
- Functional (e.g. physical, movement)
- Psychological
- None, we do not use any specific criteria at this stage
- Other

26. In order of importance, being as specific as possible, specify up to 3 criteria you use to decide a player has returned to performance

1.
2.
3.

Progression criteria during return to play following a hamstring injury in professional football

Section 5: Additional Comments

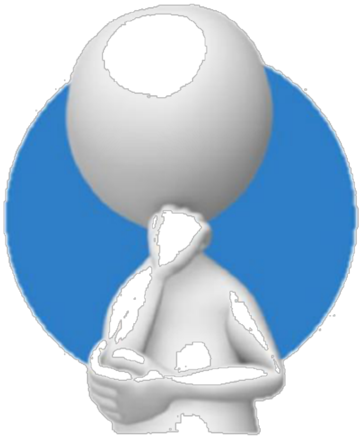

27. Is there any additional information regarding your current rehabilitation programme that potentially may not have been directly addressed within the survey that you would like to add?

Please specify below

28. Would you be open to a follow up telephone interview if requested?

- Yes
- No

29. Please provide your personal details as directed

Name

Email Address

Contact Telephone Number
